# Microwave Synthesis of Quaternary Ammonium Salts

**DOI:** 10.3390/molecules13092107

**Published:** 2008-09-03

**Authors:** Angela J. Winstead, Nicole Fleming, Krystal Hart, Deveine Toney

**Affiliations:** Morgan State University, Department of Chemistry, 1700 E Cold Spring Lane, Baltimore, MD 21251, U.S.A.

**Keywords:** Quaternary ammonium salts, Microwave synthesis, Heterocycles

## Abstract

The microwave synthesis of several quaternary ammonium salts is described. The synthesis provides comparable or better yields than published methods with reduced reaction times and in the absence of solvent.

## Introduction

Fluorescence spectroscopy has become a key technique for the detection and elucidation of biological processes. In particular, cyanine dyes see widespread application as fluorescent probes. They have been used in DNA sequencing, immunoassays, agarose gel and capillary electrophoresis staining [1], DNA analysis in polymerization chain reactions [2,3], in flow cytometry [4], or as fluorescent probes for membrane fluidity [5,6] and membrane potential studies [7]. However, common problems associated with cyanine dyes include their tendency to undergo photobleaching [8], and self-aggregation [9]. This has prompted the development of novel cyanine dyes with increased photostability, Stokes’ shift, and quantum yield for use in bio-applications. 

**Scheme 1:**
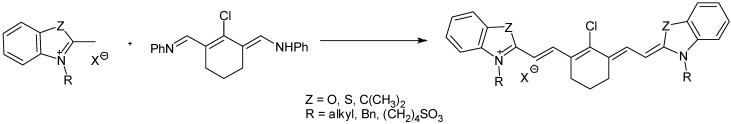
General synthesis of heptamethine cyanine dyes.

Cyanine dyes have traditionally been prepared from a condensation of an *N*-alkyl heterocyclic quaternary ammonium salt and a bisimine or bisaldehyde (Scheme 1) [9]. *N*-Alkyl quaternary ammonium salts are used extensively as precursors of near-IR spiropyrans [10] and various cyanine dyes [11]. The salts are synthesized by refluxing reagents with solvents such as chloroform, *o*-dichloro-benzene, acetonitrile and ethanol for 6 – 48h. One example requires refluxing in acetonitrile for 24 h, then treatment with diethyl ether followed by filtration. The combined filtrates are concentrated and refluxed for an additional 24 h, treated with diethyl ether and filtered [12]. This process was repeated 1-3 times to achieve the published yields (25 – 78%). Another method heats the reagents at 80^ο^C for 21 h in an ampule tube sealed with a torch [10]. Purification of the salts range from Soxhlet extraction with benzene for 24 h [13] to filtration with cold ether [12]. 

A simple efficient microwave synthesis of *N*-alkyl quaternary ammonium salts has now been developed. Reaction times are measured in minutes as opposed to hours and all of the experiments are performed under solvent-free conditions. 

## Results and Discussion

**Scheme 2:**
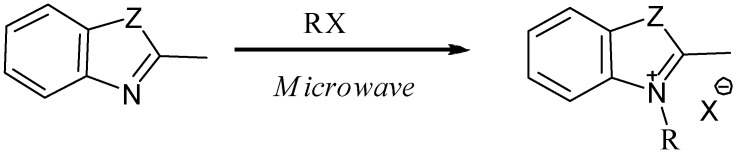
General synthesis of quaternary salts.

The reaction of 2,3,3-trimethylindolenine with an array of alkyl halides with varied functionality were studied. The reactions were performed by charging each microwave reaction vial with of 2,3,3-trimethylindolenine and an alkyl halide (Scheme 2). Our previously published reaction of ethyl iodide with 2,3,3-trimethylindolenine served as the model system [14]. The microwave reaction conditions were determined using a single-mode microwave system. The temperature was monitored throughout each reaction. The optimized reaction condition was 130 ^ο^C, ramp time: 2:50 min, reaction time: 5:00 min giving a 95% yield. 

The scope of the reaction was examined with the coupling of 2,3,3-trimethylindolenine and benzothiazole with iodomethane, iodopropane, bromoethanol, and bromohexanoic acid. The hold time, ramp time, and temperature for each electrophile was studied. The optimized reaction conditions are presented in Table 1. In most cases, the yields were comparable or exceeded the published yields. Most significant is the substantially decreased reaction time and simplicity of the reaction procedure. The yields presented are the yields without resubjection of the filtrates.

**Table 1:** Microwave synthesis of quaternary ammonium salt derivatives.

Entry	Z	R	Temp (^ο^C)	Time (min)	Yield (%)	Lit. Yield (%)	Lit Time (h)
1	C(CH_3_)_2_	Et	130	5:00	95	59	48 [13]
2	C(CH_3_)_2_	Me	110	2:30	93	75	21 [11]
3	C(CH_3_)_2_	Pr	110	7:00	83	44	24 [15]
4	C(CH_3_)_2_	-(CH_2_)_2_OH	110	7:00	73	69	24 [16]
5	C(CH_3_)_2_	-(CH_2_)_5_CO_2_H	110	7:00	59	67	12 [15]
6	S	Et	170	20:00	83	48	48 [13]
7	S	Me	120	20:00	85	60	7 [17]
8	S	Pr	170	20:00	65	5	7 [17]
9	S	-(CH_2_)_2_OH	100	35:00	58	N/A	6 [18]
10	S	-(CH_2_)_5_CO_2_H	170	35:00	51	61	48 [19]

### Work up and purification

Alkyl salts **1**, **2**, **3**, **6**, and **7** were simply filtered and washed with cold ether. The products were pure by NMR analysis and no further purification was necessary. Salts **4**, **5**, **9** and **10**, which all contain hydroxyl groups, did not crystallize right away. The reaction solution containing **4** was concentrated followed by the addition of hexanes. The solution was heated until crystals formed and then filtered. Similarly, salt **5** was recrystallized from acetone. Reaction vials containing **9** and **10** were allowed to sit at room temperature for 2-4 h, after which time crystals formed and could be filtered.

## Conclusions

The single mode microwave system has provided substantially decreased reaction times, simplicity of reaction procedure, and comparable or increased reaction yields observed for reactions conducted. 

## Experimental

### General

All microwave reactions were conducted using the single-mode Biotage Initiator 2.0 (www.Biotage.com). ^1^H- and ^13^C-NMR spectra were obtained in DMSO-*d_6_* using a Bruker Avance 400 MHz NMR and were recorded at 400 MHz and 100 MHz, respectively. All reagents and chemicals were obtained from Aldrich Chemical Company (USA) and Alfa Aesar and were used as received.

*2,3,3-Trimethyl-1-ethyl-3H-indolium iodide* (**1**): Iodoethane (1.76 mL, 0.0218 moles) and 2,3,3-trimethylindolenine (0.76 mL, 0.0218 moles) were added to a reaction vial via syringe and heated at 130^o^C in the microwave system for 5 min and a ramp time of 4 min. The crystals were filtered and washed with cold ether and dried under vacuum for a yield of 95%. ^1^H-NMR: δ 7.99-7.97 (m, 1H), 7.86-7.84 (m, 1H), 7.64-7.62 (m, 2H), 4.5 (q, *J* = 7.3 Hz, 2H), 2.8 (s, 3H), 1.5 (s, 6H), 1.4 (t, *J* = 7.3 Hz, 3H); ^13^C-NMR: δ 196.0 (C), 141.8 (C), 140.6 (C), 129.3 (CH), 128.8 (CH), 123.4 (CH), 115.2 (CH), 54.0 (C), 42.9 (CH_2_), 21.8 (CH_3_), 13.7 (CH_3_), 12.5 (CH_3_).

*1,2,3,3-Tetramethyl-3H-indolium iodide* (**2**): Iodomethane (0.44 mL, 0.00709 moles) and 2,3,3-trimethylindolenine (0.22 mL, 0.00142 moles) were added to a reaction vial via syringe and heated at 110^o^C in the Explorer microwave system for 2:30 min and a ramp time of 2 min. Crystals were washed with cold ether and dried under vacuum for a yield of 93%. ^1^H-NMR: δ 7.9-7.6 (m, 4H), 3.9 (s, 3H), 3.3 (s, 3H), 1.5 (s, 6H); ^13^C-NMR: δ 195.8 (C), 141.9 (CH), 141.4 (CH), 129.1 (CH), 128.6 (CH), 123.1 (CH), 115.0 (CH), 53.8 (CH_3_), 34.6 (CH_2_), 21.5 (C(CH_3_)_2_), 14.15 (CH_3_). 

*2,3,3-Trimethyl-1-propyl-3H-indolium iodide* (**3**): Iodopropane (0.60 mL, 0.1639 moles) and 2,3,3-trimethylindolenine (0.19 mL, 0.0329 moles) were added to a reaction vial via syringe and heated at 110^o^C in the microwave system for 5 min and a ramp time of 2 min. Crystals were washed with cold ether and dried under vacuum for a yield of 83%. ^1^H-NMR: δ 8.01-7.9 (m, 1H), 7.86-7.84 (m, 1H), 7.64-7.62 (m, 2H), 4.44 (t, *J* = 7.3 Hz, 2H), 2.85 (s, 3H), 1.88 (m, 2H), 1.55 (s, 6H), 0.99 (t, J=7.4Hz, 3H); ^13^C-NMR: δ 196.5 (C), 141.8 (C), 141.0 (C), 129.4 (CH), 128.9 (CH), 123.5 (CH), 115.5 (CH), 54.1 (C), 48.8 (CH_2_), 22.0 (CH_3_), 20.7 (CH_2_), 14.0 (CH_3_), 10.7 (CH_3_).

*1-(2-Hydroxyethyl)-2,3,3-trimethyl-3H-indolium bromide* (**4**): 2-Bromoethanol (0.52 mL, 0.008 moles) and 2,3,3-trimethylindolenine (0.60 mL, 0.004 moles) were added to a reaction vial via syringe and heated at 110^o^C in the microwave system for 9 min and a ramp time of 4 min. The liquid was concentrated using CH_2_Cl_2_. The residue was suspended in hexanes and heated, the solid scraped and filtered, then re-crystallized in chloroform. Crystals were dried under vacuum for a yield of 73%. ^1^H-NMR: δ 8.02-8.00 (m, 1H), 7.88-7.86 (m, 2H), 7.63-7.62 (m, 2H), 4.26 (t, J=5.0 Hz, 2H), 3.8 (t, J=5.0 Hz, 2H), 2.8 (s, 3H), 1.5 (s, 6H); ^13^C-NMR: δ 197.7 (C), 141.8 (C), 141.1 (C), 129.2 (CH), 128.7 (CH), 123.4 (CH), 115.6 (CH), 57.7 (CH_2_), 54.2 (C), 50.3 (CH_2_) 22.0 (CH_3_), 14.6 (CH_3_).

*1-(5-Carboxypentyl)-2,3,3-trimethyl-3H-indolium bromide* (**5**): 6-Bromohexanoic acid (0.67 g, 0.0034 moles) and 2,3,3-trimethylindolenine (0.54 mL, 0.0034 moles) were added to a reaction vial via syringe and heated at 160^o^C for 1200 s and a ramp of 150 s in the Explorer microwave system. Crystals were washed with acetone and dried under vacuum for a yield of 42%. ^1^H-NMR: δ 7.87-8.01 (m, 1H), 7.78-7.87 (m, 1H), 7.26-7.64 (m, 2H) 4.45 (t, *J* = 7.6 Hz, 2H), 2.87 (s, 3H), 2.24 (t, *J* = 7.2 Hz, 2H), 1.85-1.81 (m, 2H), 1.6-1.5 (m, 8H), 1.44 (m, 3H); ^13^C-NMR: δ 196.5 (C), 174.2 (C), 141.8 (C), 141.0 (C), 129.3 (CH), 128.9 (CH), 123.5 (CH), 115.4 (CH), 54.1 (C), 47.4 (CH_2_), 33.3 (CH_2_), 26.9 (CH_2_), 25.4 (CH_2_), 24.0 (CH_2_), 22.0 (CH_3_), 14.0 (CH_3_).

*3-Ethyl-2-methylbenzothiazole iodide* (**6**): Iodoethane (0.248 mL, 0.0031 moles) and 2-methylbenzo-[d]thiazole (0.20 mL, 0.0015 moles) were added to a reaction vial via syringe and heated at 170 ^o^C in the 20 min and a ramp time of 4 min. The resulting white solid was washed with cold ether and dried under vacuum for a yield of 83%. ^1^H-NMR: δ 8.0-7.9 (m, 1H), 7.87-7.84 (m, 1H), 7.65-7.63 (m, 1H), 4.51 (q, *J* = 7.3 Hz, 2H), 2.85 (s, 3H), 1.54 (s, 6H), 1.46 (t, *J* = 7.3 Hz, 3H); ^13^C-NMR: δ 176.7 (C), 140.4 (C), 129.3 (CH), 129.1 (C), 128.0 (CH), 124.7 (CH), 116.7 (CH), 44.8 (CH_2_), 17.1 (CH_3_), 13.3 (CH_3_).

*2,3-Dimethylbenzothiazoleiodide* (**7**): Iodomethane (0.37 mL, 0.006 moles) and 2-methylbenzo[d]-thiazole (0.40 mL, 0.003 moles) were added to a reaction vial via syringe and heated at 120 ^o^C in the microwave system for 20 min and a ramp time of 2 min. The white solid was washed with cold ether and dried under vacuum for a yield of 85%. ^1^H-NMR: δ 7.9-7.6 (m, 4H), 3.9 (s, 3H), 3.3 (s, 3H), 1.5 (s, 6H); ^13^C-NMR: δ 176.23 (C), 140.60 (C), 128.27 (CH), 127.72 (C), 127.08 (CH), 123.55 (CH), 115.81 (CH), 35.39 (CH_3_), 16.36 (CH_3_).

*3-Propyl-2-methylbenzothiazole iodide* (**8**): Iodopropane (0.75 mL, 0.007 moles) and 2-methylbenzo-[d]thiazole (0.20 mL, 0.0015 moles) were added to a reaction vial via syringe and heated at 170 ^o^C in the microwave system for 30 min and a ramp time of 4 min. The tan solid was washed with cold ether and dried under vacuum. The product was recrystallized from acetonitrile to produce a white solid in 65% yield. ^1^H-NMR: δ 8.01-7.9 (m, 1H), 7.86-7.84 (m, 2H), 7.64-7.62 (m, 1H), 4.44 (t, *J* = 7.3 Hz, 2H), 2.85 (s, 3H), 1.88 (m, 2H), 1.55 (s, 6H), 0.99 (t, J=7.4Hz, 3H); ^13^C-NMR: δ 177.0 (C), 140.8 (C), 129.3 (CH), 129.0 (C), 128.0 (CH), 124.6 (CH), 116.9 (CH), 50.4 (CH_2_), 21.3 (CH_2_), 17.1 (CH_3_), 10.7 (CH_3_),

*3-(2-Hydroxyethyl)-2-methylbenzothiazole bromide* (**9**): 2-Bromoethanol (0.44 mL, 0.006 moles) and 2-methylbenzo[d]thiazole (0.4 mL, 0.003 moles) were added to a reaction vial via syringe and heated at 100 ^o^C in the microwave system for 35 min and a ramp time of 2 min. After 4 h, a light purple tinted solid forms. The solid was recrystallized from acetonitrile and the white solid was dried under vacuum for a 57% yield. ^1^H-NMR: δ 7.62-7.28 (m, 4H), 4.2 (q, *J* = 7.2 Hz, 2H), 3.0 (s, 3H), 1.2 (s, 6H), 1.1 (t, *J* = 7.2 Hz, 3H); ^13^C-NMR: δ 178.0 (C), 141.0 (C), 129.1 (CH), 128.9 (C), 127.9 (CH), 124.5 (CH), 117.0 (CH), 58.5 (CH_2_), 51.9 (CH_2_), 17.3 (CH_3_).

*3-(5-Carboxypentyl)-2-methylbenzothiazole bromide* (**10**): 6-Bromohexanoic acid (2.9 g, 0.015 moles) and 2-methylbenzo[d]thiazole (0.40 mL, 0.003 moles) were added to a reaction vial via syringe and heated at 170^o^C for 35 min and a ramp of min in the microwave system. The solid was washed with cold ether, recrystallized from acetonitrile, and dried under vacuum for a yield of 51%. ^1^H-NMR: δ 7.87-8.01 (m, 1H), 7.78-7.87 (m, 1H), 7.26-7.64 (m, 1H) 4.45 (t, *J* = 7.6 Hz, 2H), 2.87 (s, 3H), 2.24 (t, *J*= 7.2 Hz, 2H), 1.85-1.81 (m, 2H), 1.6-1.5 (m, 8H), 1.44 (m, 3H); ^13^C-NMR: δ 177.0 (C), 174.2 (C), 140.8 (C), 129.3 (CH), 129.0 (CH), 128.0 (CH), 124.6 (CH), 116.8 (CH), 49.0 (CH), 33.4 (CH_2_), 27.4 (CH_2_), 25.4 (CH_2_), 24.0 (CH_2_), 16.8 (CH_3_).
